# The Hospital Nursing Supplement

**Published:** 1894-08-18

**Authors:** 


### "The Hospital" Nursing Mirror.

Being the Extra Nursing Supplement of "The Hospital" Newspaper.

[Contributions for this Supplement should be addressed to the Editor, The Hospital, 428, Strand, London, W.C., and should have the word "Nursing" plainly written in left-hand top corner of the envelope.]

### News from the Nursing World.

### OUR PAUPER CHILDREN.

A kind offer made by Mrs. Easton and Professor Xavier to teach swimming to pauper children at Islington has been accepted by the Guardians, and they are to have the use of the Hornsey Public Baths. This is an example which ought to be speedily followed in other districts, for healthy physical exercise is of the first importance for these poor boys and girls. The subject has hitherto claimed far too little attention, and we suppose it will be wilfully ignored until a more enlightened policy prevails. In the future perhaps the obvious economy to the state of a healthy community may be realised. In a ward, the other day, a strumous child was pointed out who lived in "The House" (situated just off a thoroughfare and guiltless of play-ground or garden). A nurse (all honour be given to her good thought) offered to have the little creature with her when she spent her hard-earned holiday with her country friends. The Board refused permission, deciding that if change were required they were the officials to arrange it. The matter dropped, and in the dull workhouse ward still may be seen the little one whose diseased childhood could be probably merged in healthy manhood by judicious and prolonged change of air.

### UNAUTHORISED "INSPECTION."

An actual, as well as a threatened epidemic increases the activity of the sanitary authorities, and induces many householders to see that their drains are in order. Probably the young man who made himself conspicuous at St. John's Wood last week was quite aware that an extra amount of inspection would be patiently submitted to, for his unauthorised visits were frequent and prolonged ones. When, however, he informed a widow lady living alone that he should come twice a day during the epidemic she began to doubt his right in the matter, and after complying with demands for money and hearing threats of fines, she communicated with the police. The plausible young man in the straw hat and canvas shoes was soon afterwards arrested, and it transpired that he had "inspected" other houses, and had obtained money in different quarters for the widow of an inspector who had lost his life through devotion to duty. The fertile imagination which had created this widow also served to evolve unpleasant smells, of which (although nobody else could detect them) he always made a note in an official looking memorandum book. Success made him over bold, and justice, in the form of a policeman, overtook him on the doorstep of the lonely lady he had so shamelessly robbed and bullied.

### UNSIGNED ACCUSATIONS.

Certain disparaging assertions have found their way into the local press regarding the nursing at the Bedford Infirmary. These complaints take the usual form of a letter, the writer of which, also according to custom, is too modest to sign his own name to it. The charges made are serious, being to the effect that a patient was not treated in accordance with the doctor's orders. Therefore the remedy was not far to seek. This nameless "Anxious Inquirer" could have had the matter cleared up immediately by application to the said doctor or the hospital committee. People possessed of grievances do not always realise that this simple course lies open before them. Mr. George Rivers has published a very kind letter in defence of the valuable institution in question, where he has himself been a patient, but as his experience was necessarily confined to a male surgical ward, it fails to deal with points raised in connection with the management of female medical cases. No doubt those responsible for the nursing of Bedford Infirmary would have given satisfaction to "Anxious Inquirer" had he made his report directly to them instead of allowing it to filter to them through the medium of the public press.

### WITHOUT BOARD.

Thirty-five pounds per annum, *without board*, is the salary offered in an advertisement for a qualified dispenser, who must be also a certificated nurse. The post she is required to fill is that of matron of a small cottage hospital, and therefore she must be a good manager, able to turn her hand to anything on an emergency. To the training and experience necessary to develop such a woman as the one indicated, should be added the study of how to live without "board." Food, raiment, and some provision for old age can hardly be squeezed out of thirty-five pounds per annum, even by a well-trained nurse with a knowledge of drugs!

### WHAT BECOMES OF THE LINEN?

We doubt if many outside people know what an important part of a nurse's work is played by the ward linen. What anxious management is required to make the stock meet all the demands upon it. The wearisome airing of damp clothes, where there is no airing closet, and then the incessant patching and mending to keep things going. And besides all this there remains the vital question, What becomes of the linen after it has been used? The nurse gets rid of her responsibility by means of "a shoot," or in the more primitive form of a tied-up bundle carried somewhere. Of course, every town hospital cannot have a laundry of its own, and the baskets in such cases are called for on certain days. There is still one institution at least where patients' things are allowed to accumulate in the basement for a whole week, and are then counted over by one of the staff and handed to the laundry maid. A more horrible task cannot well be conceived, nor a better proof of bad management. At another hospital it is common to see bundles of soiled linen strewn in front of the nurses' home. In fact, in many institutions, especially the smaller ones, the history of the linen after it leaves the wards is more startling than edifying. The carbolic tank into which everything is "shot" in some places is found useful when supplemented by a "wringer," fixed firmly to the tank. This allows of a maximum of precaution with a minimum of labour. All interested in institution management should make thorough investigation into what becomes of the linen.

### NURSES.

The Medical Officer of the Hackney Training Schools has recently given publicity to the fact that Gillespie, whose name, prefixed by "Nurse," has been so notorious, was in no way associated with the infirmary, and, further, that the nurses in the infirmary have no duties to perform in the main building, which is quite distinct from the infirmary. We may, therefore, hope that the attendants in the sick wards have really the right, earned by completed training, to bear the honourable and distinctive title of nurses.

### RESPIRATION VERSUS NOURISHMENT.

"What's the patient's temperature this evening, nurse?" "I've just charted it," returned the nurse, "and I've taken it and the pulse and respiration every two hours." The doctor looked surprised, but silently held out his hand for the neatly marked record. "What nourishment have you given?" he asked, after looking at it. The nurse seemed startled. "I'm afraid I can't tell you. I know she had some milk once, and since then some beef-tea, but I did not remember about the nourishment this evening. I was so anxious to get the pulse and respiration right." "Then perhaps I had better attend at feeding times, and see to the nourishment myself," retorted the old-fashioned doctor sharply.

### NEWTON ABBOT WORKHOUSE.

The report of the Local Government Board on Newton Abbot Workhouse has been made public, and it deals most exhaustively with the charges brought against the management there. "The evidence appears to the Board to leave no room for doubt that both the majority of the guardians and of their officers have altogether failed to appreciate the serious responsibilities that rest upon them as regards the welfare of the inmates under their care. The Board consider that beyond all question there have been cruel illusages in individual cases, and many abuses of a serious character affecting the comfort and well-being of many of the inmates, more especially of the sick, the imbeciles, and the children." This report, which is given in full in the local press, goes on to show the ways in which comforts and even necessaries of the sick inmates have been often lacking. These abuses were made public principally through the courage of Nurse Hilton, who endured discomfort and opposition until at last an opportunity was given her of speaking out. Attempts to disprove her statements failed, and she was indeed a true champion of the paupers. It is probable that her successor at Newton Abbot will have an easier experience, and she has the cordial sympathy of many friends in the good work she is taking up under the auspices of the Workhouse Infirmary Nursing Association, which has done so much to encourage the employment of trained nurses in workhouses.

### QUEEN'S NURSE AT SELKIRK.

The quarterly record of work done by Nurse Rumsey at Selkirk is very satisfactory, and this branch of the Queen's Jubilee Institute may certainly be congratulated on its usefulness. A substitute has been secured to take the nurse's duties during her annual holiday, which is an admirable arrangement not always found feasible in country districts.

### VALUABLE AUXILIARIES.

District nursing among the very poor can only be accounted successful when the nurse's skilled labour is supplemented by an adequate provision of nourishment and raiment. Not only the doctor and nurse, but others of the general public share in the good work. Every district needs, and many have, a Samaritan or similar society which helps to provide the needful "kitchen physic." Needlework Guilds, too, are of immeasurable value, and in connection with these may be named the St. Patrick's Guild, associated with the Home for Nurses in Dublin, affiliated with the Queen's Jubilee Institute. Its object is to provide the nurses with garments for their patients during illness and, if needed, on recovery. Sheets, blankets, and pillow cases are always useful, and information as to the special wants will be gladly given, and subscriptions received, by the Lady Superintendent or the Hon. Secretary, 101, Stephen's Green, Dublin.

### CARRICKMACROSS.

It is proposed that two nuns shall be engaged as infirmary nurses at Carrickmacross Workhouse. The Superioress of the Convent of St. Louis is willing to supply two Sisters at £30 per annum each, but we are not at present informed whether these ladies are duly trained nurses, and if they intend to include night nursing in their duties. It is to be hoped that the proposed arrangement will not be confirmed without due consideration being given to these two very important matters.

### GENUINE NURSES.

In spite of the alarming prognostications of a few alarmists, we do not ourselves imagine that any true nurse need fear "degradation" of her "profession" resulting from the recent mushroom growth of "sham nurses." The latter are too poor an imitation to deceive anyone. Happily they do not attempt to copy the quiet, modest nurses, whom we recognise and honour instinctively. The impostors only take off the worse type of nurse, exaggerating her follies of dress and manner. If the public fails to discriminate between the shades of dyed hair and the "tinted" cheeks, it is individuals and not uniform which deserve blame. The wise woman who makes her dress a mere harmonious accessory of her calling is not likely to be injured by caricaturists, about whom she seldom troubles to think.

### SHORT ITEMS.

At Grangemouth a meeting was recently held, under the presidency of Lady Zetland, for the purpose of establishing a nursing association in the town.—The Duke of York has consented to become a patron of the Seaford Convalescent Home.—Miss Rose Petty, Miss Dean-Pitt, Miss Ellison, Miss Pierson, Miss Taylor, and Miss Maud Miller are amongst the students who successfully passed the June examination in Physiology and Hygiene, in connection with the Science and Art Department at South Kensington, and Nurse Agnes Mackie has passed in Physiology.

### On General Nursing.

By ROWLAND HUMPHREYS, M.R.C.S., L.R.C.P.Lond.

XXVI.—TYPHOID FEVER (*continued.*)

THERE is no very reliable sign of impending perforation. When it has taken place it is shown by symptoms of collapse, and usually by pain, small pulse, and distended stomach. Sometimes the symptoms are so slight that a little coldness of the extremities or of the tip of the nose is the only sign. The only hope for the patient in a bad case of perforation lies in a rapid abdominal section and closure of the wound in the intestine, or bringing it to the surface of the abdomen and fixing it there, followed later on, if the patient survive (of which there is small probability), by an operation for restoring the continuity of the bowel. Immediate operation is required, taking care that the temperature of the patient is not lowered during the operation, and keeping the strength up as best one may.

The fatalities, just mentioned, may occur either in the third or subsequent weeks.

Hypostatic congestion of the lungs is of common occurrence. So also is bronchitis. These complications may occur at almost any stage of the complaint, and are treated in the ordinary way, with stimulants, cotton wool round the chest and a steam-tent. Blueness of the lips, &c., may be the only sign of the condition of the lungs; careful note of the patient's appearance from day to day must therefore be taken, and the attention of the medical attendant called to any alteration.

Ulceration of the larynx is said to occur in all cases, but it is not often possible to absolutely diagnose it, as the state of the patient precludes laryngoscopic examination on account of the consequent fatigue.

Meningitis, and even mania, sometimes occur in the course of the disease, and are very fatal.

Inflammation of the veins of the legs may occur, and the clotting, which is the inevitable consequence of it, may extend up the limb to the large abdominal veins and cause death.

During the fourth week the temperature chart shows a decided alteration. The temperature varies several degrees between night and morning, and is lower as a rule, and the condition of the patient improves slowly; the great prostration passes off, the diarrhœa ceases or lessens, more food is taken and the patient talks more clearly and more connectedly.

One of the great troubles in typhoid is to keep the mouth in a comfortable condition. The tongue is always more or less thickly coated at the commencement of the illness, and as the case goes on it tends to become dry and brown. This is one of the signs of great exhaustion, and indicates that necessity for the administration of brandy. This drug should be given at two-hour intervals, and in quantities proportioned to the state of the patient, and to the age and habits, if an adult. Some patients, notably those who have been in the habit of taking a good quantity of alcohol daily, require and can take a large amount. The amount varies with each patient, and is best gauged by the tongue becoming soft and moist, and remaining so. If, while taking alcohol in large quantity, the tongue becomes dry, this indicates a less dose, and other indications in the same direction are increased, or excited pulse, excited brain, restlessness. Alcohol acts in several ways; as a stimulant, as an antiseptic, as a sedative; and the effect sought after must be experimentally obtained and then kept.

In order to wash the system out a large amount of fluid should be administered, and the liquid so taken may be used as a vehicle for extra food or for additional acid.

The internal administration of antiseptics has been alluded to, and the drugs used for this purpose are numerous. One rather favourite drug is chlorinegas in a state of solution. Another drug is naphthaline, another β-naphthol, another salol.

The effect of these drugs is to diminish the foetor of the stools, in itself no small gain; the tongue gets cleaner, the fever lower and more easily brought down, the disease is not uncommonly cut short, and the convalescence made more rapidly. Calomel is another excellent antiseptic, and is often given at the outset of an attack in the hope of cutting it short.

For the diarrhœa injections of starch and opium (20 or 30 minims of laudanum and 2 oz. of starch), or of tannic acid, 10 or 20 grains to 2 oz. of thin mucilage, with a few grains of Dover's powder, are excellent. Where the diarrhœa is troublesome the beef-tea as ordinarily made by boiling should not be given, as it is an excellent medium for the growth of the various bacteria found in the bowel, and, after administration, fermentation, the result of their growth, is rapidly set up.

The high temperature, which is often present, tends to weaken the heart, already in considerable danger from the fatty degeneration of its muscle, which occurs in all cases. Immediate action must therefore be taken to reduce the fever whenever it is above 103° F. The simplest means of doing this is the use of hot sponging, the water used being about 90° F. in the basin. Each quarter of the body is sponged for some minutes, the chest and abdomen first, the back afterwards. The temperature continues falling for some little time after the cessation of the sponging; it should, therefore, not be reduced below 101° F. by the process. Cold sponging, or sponging with iced water may also be employed, or the ice pack, consisting of ice broken in small pieces between two layers of a sheet, and wrapped round the body and chest. Another means of reducing the temperature is the ice-cradle. Iced rectal injections have also been employed, and iced food (such as beef tea in the form of an ice) may assist. Leiter's irrigating tubes are at times very useful; it is better to use ordinary cold water in them and not iced water.

The result of the cooling process is not only the immediate one of bringing the fever down, and so reducing the waste of the tissues and the fatty degeneration of the muscles, and the injury to the nervous system, but, in addition, the cerebral symptoms diminish, the pulse improves, and the patient takes food better, and is altogether happier.

The cold bath should have been mentioned amongst the refrigerants. Recently the experiment has been tried of keeping the patient constantly in a tepid bath in which the water was from time to time renewed, and was kept continuously at the same temperature; but the more ordinary procedure is to place the patient in a tepid bath and allow the water to cool slowly, gently rubbing the patient while in it.

To turn from the nursing side of the question to the ætiological one, there is no doubt but that every case of typhoid fever proceeds directly from a previous one, though the connection cannot always be traced. It is advisable for the nurse to look into matters for herself in a quiet way, as she may thereby not only protect her own health, but also may be the means of preventing an epidemic through the house.

The disease is most often infectious through soiled bed-linen. The soiled article does not become infectious till some twelve hours after soiling, but it then becomes highly infectious, and any neglect on the part of the nurse will, therefore, expose her to the disease. It is necessary, therefore, to place all soiled linen at once in a bath containing either 1 in 50 Jeyes' fluid, or 1 in 20 carbolic acid, or other disinfectant, and to leave them there for at least twelve hours. The drains in the house should be well flushed out with two or more pails of water and disinfectant each day.

After the disease has apparently run its course a relapse is not uncommon, and this is especially likely to occur if any improper food is given. A relapse runs just the same course as a first attack, and though it usually occurs within five or ten days after the first one, yet the patient is liable to it for some considerable time after apparent recovery.

In children and occasionally in older people, the disease runs a shorter course than usual.

Another form of the disease is where the temperature runs downwards instead of upwards, forming an inverted chart. In such a case there is always great difficulty in persuading the patient to take the proper precautions, and he may become what is known as a case of ambulatory typhoid, that is one which goes about in the ordinary way spreading the disease as he goes.

### Structure and Care of the Teeth.

### II.—VARIETIES OF FORM.

The second teething is effected with some few and slight modifications, in a similar way to the first. The shedding of the first set of teeth arises from the absorption of their fangs. The nature and structure of the dental tissues themselves may be stated in general terms to be three in number—(1) dentine; (2) enamel; (3) cement—and these substances in health are all but indestructible. By these it is possible to identify animals, and by them the scientific man has to recognise those stupendous creatures that peopled the earth even before man existed. By observations of the kind and form of the teeth much can be discovered respecting the nature and habits of their possessor. The form of jaw and head of the animal to which the tooth belongs can be decided, and it can be ascertained what is the structure of the spinal column and other bones, which alone are consistent with the shape of a particular skull, and, finally, the kind of extremities required by such an animal are thereby indicated. Hence, through one tooth the whole skeleton can be built up. As an instance of this it is said that after an anatomist had so built up the skeleton, from his imagination, as it were, since he had no part of the animal before him except a solitary tooth, later on, during more extensive excavations, the complete skeleton of the same animal was found, and it was discovered that the imaginary skeleton was correct. The tissues which form the teeth are of the same general nature in every animal, although in some cases one or other of them may not be present.

Dentine, or the body of the tooth, is composed of a vast number of extremely small tubes embedded in a foundation of material formed of lime salts so that it resembles bone. These tubes are arranged so as to lie with one end opening towards the pulp at the centre of the tooth, the other ending at its external surface, and each measures about the ten thousandth part of an inch in diameter. Their use is to maintain the life of the tooth by securing nutrition from the central cavity running along each tooth, which contains what is known as the "pulp," a substance composed of nerves and blood vessels, which are needed to nourish the tooth.

Enamel consists of a collection of dense flint fibres or columns closely packed together, and standing on the surface of the dentine where the tooth is exposed in the mouth, and for which it forms a mechanical and protective covering.

Cement, or the substance covering the root, closely resembles true bone, and acts in the double capacity of assisting the central canal to give nourishment to the dentine, and as a bond of union between the root of the tooth and its socket in the jaw.

Wherever any one of these three substances is weak or defective they are very liable to decay on account of the very small amount of vitality which they possess at the best of times. When bone becomes diseased and part is separated, it can be repaired by the addition of fresh bone matter brought by the blood. If, however, our teeth decay they are not renewed, but require a dentist's filling to make up.

The teeth are placed in very trying surroundings, exposed constantly to damp and change of atmosphere according as we keep our mouths closed or let in cold outside air when we speak. When we eat, particles of food remain behind, and are changed by the action of the saliva. Moreover, we work our teeth very hard, and hence, though in an absolutely healthy condition nature has provided them with the means of resisting all harm, if the general health is bad, disease is present, or there be a flaw in any one of the tissues, especially in the enamel, the effects of this and the evils to which it is exposed will speedily end in the death of some portion of the substance forming the tooth. Decay follows, and the central canal, with its extremely sensitive, nervous contents, is exposed. Acute toothache results. Sometimes the cement is the seat of an overgrowth of its substance, owing to some irritation or inflammation, and this overgrowth pressing on the nerves of the pulp causes great pain of a neuralgic character. Similar pain is felt by bony growths in the central canal, which press on the pulp in the same way. Sometimes the soft substance outside and distinct from the tooth substance, which keeps the tooth firmly in its socket, becomes inflamed, and then there occurs gum-boil. These and other troubles with the teeth depend greatly on and are influenced by the general health of the individual, as well as on local and more direct causes. The teeth of man are only one of many kinds throughout the animal kindom, and give a very poor idea of the variety of teeth that are found, both as regards number, form, position, uses, and functions. Thus fish have innumerable teeth, whilst the "narwhal" or sea unicorn, found in the northern seas, only possesses a solitary tooth, in shape like the unicorn's horn, as seen in heraldry. Man has his teeth limited to his mouth and jaws, but in fishes and reptiles they are scattered over all the bones of the mouth, the tongue, and the gullet; and in the sea-urchin the jaws themselves answer the purpose of teeth. In crabs, beetles, and some insects we find what answers the purpose of teeth in the stomach.

Again, as regards form and size. In some of the ray and skate tribe of fish the teeth are simply flat plates, whilst in the walrus they form a tusk, and in the whalebone whale they form plates of whalebone. In size they vary from minute points to huge tusks which have been known to weigh 200 lbs. and measure 11 ft. in length in the mammoth. Then as regards their use. In the lion and other similar beasts they serve as weapons of attack or defence. In the beaver they are the means of building; the walrus uses them to climb by. Some sharp-toothed birds, now extinct, used them to secure their prey; fish use them for seizing and holding; serpents for poisoning; and man uses them for preparing food, for speech, &c. We must next consider what can be done to preserve the teeth, and especially those of children.

### A Case for Assistance.

Bearing a double burden of illhealth and poverty, the nurse whose cause is pleaded by Miss Bell and Miss Farlow yet has hope of supporting herself by means of the little shop, which still wants stocking. Her friends think that £20 would suffice, and would also clear her from debt. They believe, too, that many readers of The Hospital will help this afflicted fellow nurse to retain her independence and to make a modest livelihood. Offers of help and subscriptions will be gladly received by Miss Bell and Miss Farlow at the Liverpool Homes for Aged Mariners, Egremont, Cheshire. They gratefully acknowledge 5s. from Miss Lucy White (second subscription), and 2s. 6d. from a "Cornish Nurse" (Helen), making £4 13s. up to present date.

### Where to Go.

A sale of work in aid of the funds of the Midwives' Institute, 12, Buckingham Street, Strand, will take place in December. Contributions of useful clothing and other articles will be gladly received and acknowledged by the Hon. Secretary.

Church of England Waifs and Strays.—Mrs. Trickett's annual sale of work for this society will take place in November. Contributions should be sent to Mrs. Trickett, 38, Minford Gardens, West Kensington.

### Our American Letter.

The accounts of Miss Hampton's wedding in The Hospital have interested us much, for Miss Isabel Hampton's work and her admirable book have combined to make her personality one of special interest in the American nursing world. Indeed, it seems hard to realise that she has ceased to be the guiding spirit of the training school with which her name is so closely connected at Johns Hopkins Hospital.

In this month and next quite a number of our graduates, and probably a few superintendents of training schools, will be in Europe, for we notice each year that more of our nurses esteem a long holiday as the natural sequence to their training, and very wise it is of them to go and see the hospitals and nursing homes of other countries before settling down to their life-work.

As regards news here there are various symptoms of encouraging progress to report. Last month the Fort Dodge Hospital, Iowa, was opened, and a pleasant fête marked the occasion. A special point in connection with the new hospital may be cited for imitation, namely, the furnishing and fitting of the five general wards by "The King's Daughters," "[illegible]e Catholic Ladies," "The Women's Auxiliary," "The Children's Hospital Corps," and one by Mrs. Duncombe. The work has been admirably carried out, and nothing has been spared to make each ward as complete as possible, and much friendly emulation has no doubt existed. The hospital is open to all who need medical treatment, but "particularly for those who are homeless." The attendants at Ward's Island Insane Asylum recently sent in a complaint, signed by eighty-four of their number, respecting the food supplied, and it is likely that the inquiry which has been made into the management of the institution will result in benefit to both patients and staff.

Considerable progress has been made by the Alumnæ Association of the New York City Training School, and the receptions are well attended by members and their friends. The Alumnæ Association is connected with Worcester, City Hospital Training School, and has celebrated its first annual meeting this summer.

It has been decided to establish a nursing training school in connection with the Samaritan Hospital, Sioux City, Iowa, and to give the graduates a two years' course, commencing from October next.

Mrs. Clara Weeks-Shaw, whose text book on nursing ought to be in every nurse's hands, was present at the graduating exercises of the Paterson General Hospital Training School, when a particularly interesting gathering took place. In fact, the past season has been specially marked by the successful character of these meetings.

### Queen Victoria's Jubilee Institute for Nurses.

Notice has been received that the following Scottish nurses have been placed on the roll of Queen's nurses: Nurses Beatrice Graeme and Katharine S. Macqueen (Perth), Catharine A. O'Sullivan (Glasgow), Elizabeth Thomson (Annan), Hughina Fraser (Greenock), Barbara Biggart (Kirckaldy), Isabella Bayne and Isabelia M. Davidson (Paisley), Rebecca Camp (Moniaive), Annie L. Day (Aberdeen), Cecclia B. Fairley (Johnstone), Mary F. Wade (Peebles), Alice M. Rumsey (Selkirk), Emily M. D. Walton (Beith), and Annie L. Steen (Port Glasgow) Twelve of these nurses were trained in the Scottish District Training Home, 29, Castle Terrace. Nurse Bayne was trained at Paisley for the staff of that branch; and Nurse O'Sullivan has been placed on the roll on account of satisfactory service to the Glasgow branch prior to application.

### Notes from Germany.

Strict regulations have been put into force in Germany to prevent a spread of the cholera which has again made its appearance. These regulations are most stringently carried out around the Russian frontier, the coast, and in Berlin. It is not anticipated that the country will be severely attacked this year, as Hamburg is now in a good sanitary condition, and most other German towns are in too healthy a state to allow of the spread of any epidemic. This was clearly proved in 1892, when, with the exception of Hamburg and Altona, which adjoins it, no other town was severely stricken by the epidemic.

An army surgeon, Dr. Heyse, of Berlin, gives a full and detailed description of the construction of several portable hospitals in one of the leading medical journals this month.

The floor of the Doecker Hospital is so practical in its formation that it deserves special mention. All the parts are packed for transfer in ten or fifteen packing-cases; the latter, on arrival, are taken to pieces and form the floor. Each piece closely fits the other, and feet are placed beneath to raise it from the ground. The complete weight of the hospital, including domestic offices, is 8,700 kilogrammes, and can be borne on two two-wheeled conveyances. Many of these portable hospitals are now being made with sail-cloth, which is hooked to the wooden frame, sufficient space being left between the frame and the sail-cloth to allow the air to pass. Heating is usually done by means of iron stoves. Besides the special "Doecker" construction, there are four others authorised by the Prussian Minister of War. They are:

1. The hospital designed by Dr. Zur Nieder.
2. The Bernhardt Grove Hospital.
3. The hospital of Vogler and Noah, Hanover (of sheet-iron).
4. The American (Ducker).

These can all, if desired, be used as shelters for workmen, &c. No hospital that I have ever visited has pleased me so much as the Stadt Kraukenhaus, in Leipzig. It is an immense building, one of the most important in Germany, alterations and improvements being continually made. The front ground floor is occupied by the officials and medical staff. The staircases are very wide as well as the corridors, the dispensaries are at the back of the building, and the fever and the cholera wards are detached from the main structure. The hospital provides accommodation for over 800 patients. Each bed runs on castors, and stands about a foot from the wall. Connected with each ward there is a bath-room, a comfortably furnished room for the charge nurse, and a separate small room for particularly severe cases. A corner of the ward is screened off for bandaging and minor operations. The wards, heated by one or two stoves (according to the size of the ward) standing in the centre, are as spacious, airy, clean, and comfortable as the most exacting patient could desire. It is a pity that the sisters and nurses do not wear a uniform, instead of dressing indiscriminately in grey, dark blue, brown, or black dresses, but all wear the ugly, large white cap, almost concealing the hair. However, their nursing powers are said to be of the highest degree of excellence, though their hours on duty are much too protracted.

What struck me most pleasantly was the large, wide verandah extending along the sides and back of the building. The patients in summer are wheeled out here to enjoy the lovely garden, the sunshine, and the fresh air, which are real luxuries in the centre of a town with over 370,000 inhabitants. The medical staff is an excellent one, and numbers of students after passing their "final," flock to Leipzig to gain further experience before beginning a practice of their own.

R.B.

### Notes from India.

(By a Correspondent.)

There is little or no nursing news to record. Enteric fever has been bad as usual, and the rainy months are causing many fresh cases. Private nurses are getting a little more employment, and for midwives, as usual, there is plenty to do. I have had the greatest difficulty to find one for a friend, diplomaed nurses asking 200 rupees a month and travelling expenses paid, and others, qualified as monthly nurses only, 150 rupees and expenses also. In despair I wrote to a sergeant's wife who had experience but no training, and she would attend the confinement for 30 rupees, but if her services were wanted afterwards they would be 5 rupees a day. At the last moment an old nurse I had befriended in days gone by said she would take the case, but she would have to bring her baby with her, who would be then two months old. She is a splendid nurse, with excellent diploma and certificates, and is going to take my friend's slender income into account.

As I have nothing to tell you about nursing, the Sipi Fair may be interesting to some Hospital readers, and it certainly is a quaint remnant of barbaric times.

The Sipi Fair was held this year on May 14th; it had not taken place for seven years. It is a sight to see once in a lifetime, but not a thing to be attempted a second time. Towering above Simla are the Himalayas, and stretching far away in the background rise peak upon peak, till we see in the far distance the perpetual snows. Eight miles from Simla is Mushobra, the suburban retreat of the Simla stars, where they take refuge occasionally from Saturday till Monday, Sunday being devoted to picnics, luncheons, and other gaieties. Here are pointed out the villas of His Excellency the Viceroy, and Lord Elgin, a worthy representative of the Scottish Kirk. The beautiful grounds of Kenilworth, extending over 14 acres, belong to His Excellency Sir George White and Lady White. Crowning a summit is the picturesque residence of Pelliti, pastry cook, hotel keeper, &c., one of Simla's celebrities, as great a man in his line as the worthy generals and members of Council who occupy the other bungalows. Below the Mushobra Hill lies the Sipi Valley, and unfortunately for those who go to the famous fair it is held at the bottom of the other side. Invitations were issued to all the European society of Simla by Sir Edwin Buck and Sir Charles Pritchard to a picnic breakfast at Mushobra from half-past nine till half-past eleven. The day rose hot and thundery, and we had to get up very early to be ready to start on our wild and interesting way. The jin rickshaws or bath-chairs were propelled by the domestic liveried coolies (Jampani), or by the Jungli coolies for less wealthy travellers, with bare limbs and bodies, and dirty cloths girt about their loins. All were soon spinning out of Simla—four men to a rickshaw, as a rule, but most people were merciful and took five to Sipi. These men go at a great rate. I have been driven nine miles in an hour and twenty minutes, and the last mile all up hill.

About 400 of these conveyances were wending their way to Sipi. Some people walked, others went on horseback, and so with one continual stream along the narrow road the crowd went on. Down the Mall, round Jakks, where the famous monkey temple is, through a native bazaar, then by a tunnel right through one of the hills, out at the other side along the side of the bare hill side, till we came to the Octroi, then the scenery was lovely, the deep wooded khuds below us, and the rocky-wooded banks above, on to the Mushobra Bazaar on the top of a plateau. The picnic was held on the side of another hill, so up we went over carpets of maidenhair fern, spiræa clumps, not then in bloom, to a natural terrace or ledge. There, with all the youth, beauty, rank, and fashion of Simla, we breakfasted.

After breakfast we followed the crowd down some hundred feet to the Sipi Valley. The rickshaws descended a steep zig-zag footpath; the heat and dust were frightful, and the horses, who were wedged in between the rickshaws, caused general confusion by occasionally rearing and kicking. Returning up hill one horse kicked my friend's Jampani in the chest, they backed her rickshaw into mine, and there was a general bumping down the hill some distance till a horse and its rider went over the side of the path and rolled a short distance down the khud. Sipi belongs to the Rajah of Koti, and the hill tribes, with a scattering of Thibetans, crowd to this fair. It is a kind of hiring fair for them, in fact, a matrimonial market. Here the would-be brides of the hill tribes come to be sold in marriage, and in their bright and tawdry costumes they form one of the principal features of the fair. They all sit in an enclosure grouped on the side of the hill, looking as if they quite enjoyed the whole thing; they did not mind being stared at by the group of natives and Europeans who surrounded them all day.

Most of the fair dames wore tight trousers, and wore sarahis or veils of white, majenta and gold, green and gold, deep purples and gold, yellow, rose, pink, and every piece of crude colouring that could be thought of were jumbled up, yet forming a most harmonious whole, their spangled jewels flashing in the sun. Necklaces, head and brow ornaments, nose rings, ear-rings, not in pairs, but in dozens, thumb rings with a little mirror on, and toe rings, armlets, bangles, and anklets. Many of these though dusky were handsome and beautiful. The older women try to drive a trade with their jewels, for which they ask enormous prices from the English visitors. One hideous old woman in a dirty red cloth head-dress, covered with huge rough Thibetan turquoises, of no marketable value at all, asked 300 rupees for it.

Some of the women wore curious silver chains, handsome enough in themselves, but decorated with all kinds of odd things, such as the old brass ornaments off horses' blinkers, pike spoon, bent rupees, and broken bits of second-hand ornamentation.

An old woman who was especially admired wore red trousers, a dirty white shirt, a blue overcoat, and her hair plaited and twisted through a dirty black horse-hair wig; the plaits were mixed with horse-hair tied with red braid, and stuck out in horns all round. On the top she wore several brass and silver ornaments stuck in a red cloth head-dress, about one yard long and six inches broad; it commenced at her forehead and hung down behind past her waist; this was also studded with large, various shaped, very hideous green turquoise. Like the others she wore heavy loose rings, a large bangle with spangle and tinsel fringe in her nose, and her ears were studded with silver ornaments round the rims.

Photographers, amateurs and otherwise, were busy all over the place, but the Thibetan and hill beauties refused to be photographed, except for a consideration.

There were native merry-go-rounds that turned anyone's brain to look at. The jampanis and coolies were rapacious in begging for "backsheesh," all of which quickly disappeared in the Government grog shop, or in the delights of those whirling wheels.

There were a number of booths, all the wares spread on the ground, some Indo-Birmingham jewels of tin and glass, a few the real thing and country made, for which were asked fancy prices. There were Hindu rosaries of peach stones, wood, and sandal-wood berries.

At these booths the sons of Mars, Apollo, and Mercury might be seen decking their Venuses and Junos with these paltry bangles, beads, and necklaces, perhaps on the top of a gown from Worth's or Creasy's. Then there were stalls of native sweets, and the Government grog shop presided over by a native policeman squatting on two barrels.

The Viceregal party had a tent in which they entertained several of the European visitors at lunch, those not so honoured picniced under the trees.

The Rajah of Koti arrived on his state elephant, whose face was painted vermilion and blue in a curious pattern, the howdah it carried was draped with red. The fair visitors availed themselves of the elephant for rides *à la* Zoo. The Rajah had a chamiana, or sideless tent, to which he invited everyone to tea. Lady Macdonald invited everyone to a picnic tea at Mushobra, where they could rest and be refreshed after the trying ascent of the hill. But the thunder-clouds had been threatening all day, and at four p.m. they burst, and down came the storm. Heaven's artillery thundered and crashed through the hills, and so ended the Sipi Fair of 1894. Fortunately I had left at half-past one and so escaped the storm, but never, even as it was, shall I ever forget the fatigue of the day, the overpowering heat, and the sickening smell of glue and black humanity. Thankfully I crept into my bed at four p.m., but did not escape influenza any more than many others who were visitors at the Sipi Fair.

Coming home I passed two little Goorka soldiers marching along with two shy little brides, who kept to their own side of the road and looked demurely on the ground.

### Holidays in Japan.

(By Sister Norma.)

### I. MIYANOSHITA.

Miyanoshita is the favourite holiday resort of the foreign residents in Yokohama. It is within an easy day's journey, and transplants us from the busy commercial seaport into a purely Japanese mountain life. The paper village, with its fantastically thatched roofs, lies concealed in the gorges of the Hakone Mountains, which are the foot hills of the sacred Fujiyama. We left Yokohama about half-past ten p.m. by train for Odawara, the first stage of the journey. Perhaps one of the most entertaining sights in Japan is the railway station, and as we had ten minutes to wait after taking our seats in the diminutive carriage, plenty of amusement was afforded us in watching the crowd of holidaymakers shuffling along.

Nurse Edwards and I had determined to spend a well-earned holiday in Miyanoshita. We had had a hard winter engaged in fighting against influenza and scarlet fever, but now, with the coming of the warm weather, our little hospital was comparatively empty, so we started off with light hearts to accept the gifts of the gods.

What a crowd came hurrying along. Japs, Chinamen, Englishmen, Germans, all leaving busy Yokohama to fulfil very different ideas of enjoyment! The English and Germans mostly go to Miyanoshita to enjoy the mountain air, and indulge in a course of the luxurious sulphur baths. The Chinaman, scorning eagerness or hurry, sails past, magnificent in his holiday costume, a delicious mixture of blues, pinks, greens, and mauves, with his hands hidden up either sleeve. If he misses the train he intended to catch, for all the annoyance shown on his stoical countenance, it appears evident that the next one will suit him much better. In the jostling crowd he moves not one inch to the right or left to oblige anyone. His one idea of pleasure is gambling, and he is now on the way to some little "hell" of his own, where the combined vices of opium and gaming will convert this respectable-looking merchant into the veritable "Heathen Chinee." The gentle Japs push along, some of them, in a ludicrous mixture of cheap European clothes, much impeded in their progress by heavy German boots, yet by far the greater proportion (though Yokohama is one of the most advanced cities in Japan) in their native "kimonos." Our voices are deafened by the scruf, scruf of their wooden clogs along the platform, and the pretty little women and the shabby little men are more than usually made up of bland smiles. They carry their "chow" (food) in white wooden boxes tied up in blue cotton cloths, and the whole family is there from the grizzly-bristled great-grandfather, marvellous in his ancient ugliness (for I cannot agree with Mr. Clement Scott that old age only is beautiful in Japan) to the infant great-grandchild slung on its child-mamma's back. They go out to the cherry orchards at Kame-ido, and will spend the entire day sitting on their knees on bright scarlet rugs spread below the cherry trees feasting their eyes on their beloved Sakura (cherry blossom) and refreshing the inner man with the contents of the numerous small boxes stacked one on the top of another so that each may form a lid for the one below. Their food consists principally of rice and tea, varied with a multitude of sauces and a little cold fish. It seems strange in a land of such extreme poverty that the love of beauty is so strong in these artistically-reared people that they gladly devote their time and land to the growing and training of these cherry and plum trees, which yield absolutely no fruit. Probably one of the party will read aloud from their ancient classics, or one who is a poet himself will compose and write in his exquisite penmanship a delicate ode to his favourite blossom, and hang it on a branch of the trees, for there is a true "poet's corner" in this garden of pink blossoms.

But I have digressed from my point, which was to be health-giving Miyanoshita. The sight of these smiling, dainty people diverted my thoughts to themselves and their favourite pastimes. They are so gentle and clean, and delicately dressed, a swaying mass of soft grey and blue kimonos, and glossy blue-black hair. They may have vulgar minds in their beautifully poised heads, and we know that faces "are not always what they seem," but what a different sight would the platform of a central station present in our own country on a bank holiday, though in Japan probably not one in the well-mannered crowd belongs even to the middle-classes.

We are off at last, and the platform is still a mass of smiling figures, bowing farewell to their more fortunate friends. There is a great deal of hissing and rubbing of knees, and "sayonara" (good-bye) is called out over and over again. It is a word that always suggests a great parting and sadness, and with it is borne along the lighter, heartier "auf wiedersehn." The Japanese women folk scramble up on to the seats—some native fashion sitting on their knees—others, though terribly ill at ease, conforming to the European custom. All have taken off their clogs, and even the ones advanced enough to wear boots made in Germany are breathing freely again in their stockinged soles.

### Lepers at Robben Island.

Quite an exciting scene is reported to have taken place at Robben Island on the occasion of the recent visit of the Colonial Under-Secretary. The discontent seems to be connected with the Leprosy Commission, especially with the statement regarding "the discharge of self-cured cases." The disturbance was somewhat formidable, the poor secretary, Mr. de Smidt, being mobbed by the lepers. The police endeavoured to disperse the crowd by firing their revolvers over the heads of the people. Unfortunately a male leper was wounded. Peace was eventually restored by Mr de Smidt promising that their grievances should be inquired into. The picture of a turbulent crowd of these afflicted men and women forms a pathetic contrast to their normally peaceful condition, of which a description was given in The Hospital last October by one of our South African correspondents.

### English Nurses on the Labrador.

(Communicated.)

On June 7th the good ship *Albert* set sail for Labrador, taking the doctors, the two English nursing sisters who were out last year, and a good supply of comforts for the sick on that lonely coast. The work done by the hospital branch of the Mission to Deep Sea Fishermen is comparatively little known. Although many readers of The Hospital are doubtless acquainted with fishermen nearer home, and even with the actual boats in which they leave the harbours on the East Coast, yet few can realise without actual experience the sickness, bitter cold, and loneliness endured in the pursuit of fish for the English market. Until the visit paid to Labrador two years ago by a mission ship, still less was known of the terrible hardships suffered out there. Last year some articles in The Hospital gave interesting information about the noble work awaiting accomplishment on that coast, and some account of this year's expedition will be equally welcome it is hoped to English nurses at home.

Before leaving Swansea all the crew were entertained at a sumptuous tea, followed by what one of the guests described as "a real joyful time." Photographs were taken and many good wishes interchanged for a successful winter's work and a safe return. Early on the morning of June 7th, all were on board and sail was set for Labrador. Swansea Bay and The Mumbles made a fine picture, which was regarded with longing eyes as it gradually receded from sight.

Very rough weather and constant sea sickness were the chief characteristics of the voyage, doctors and nurses alike suffered, and the cheery cry of "land ahead," on July 1st, was indeed a most welcome sound. An hour later this announcement was followed by another: "Field of ice sir, as far as we can see; bergs all around!"

Then we went north for some sixty miles, but the captain could see no way of getting through the ice and landing. In some parts the ice is twenty miles in depth and the further we went the worse it got. The "field" instead of being as might be expected, quite smooth, is very rough and "hummocky," with huge bergs jammed in here and there. The bergs this year seem larger than those we saw last season, and the colours even more beautiful. One special berg was enormously high and of brilliant blue, and others were pale blue and green. There were numbers of "ice birds" resting on the bergs, but they were hardly distinguishable, being themselves quite white. Failing to find a passage through the ice to the north, we went south next day, but had no better success. The ice up and down the coast being much broken up, quite a roar was to be heard as the pieces grated sharply together. At the same time, on the lee side of the ice, the water lay smooth as a lake for miles, and the *Albert* was as quiet as if in dock. We made several passages through some parts of the ice, but could not make much headway, and finally the ship got so jammed in amongst the ice during the night that the sails were lowered and made snug, as nothing could be done till morning. Very early the captain saw that the ice had left the shore, and he made out an opening. For an hour and a half, in spite of the intense cold, he remained at the mast-head, giving orders which were carried out with marvellous rapidity. At last, with a loud crash, we got through and into the open sea in spite of a thick fog which had come up. When this cleared the mirage prevented our making the land, and it was not till the morning of the 5th that we saw Battle Harbour.

The people on shore soon recognised us and hoisted a flag, and then a boatful came out to greet us. Everyone here considers it very wonderful that our vessel came through the ice as she did; it proved her to be a fine boat and the captain to be an excellent and experienced one. When we got near to the harbour we found lots of flags flying, and all the people standing at their doors waving handkerchiefs and even aprons.

The general rejoicing at our advent was most touching, some of the poor women actually weeping for joy. The first business was to get the little hospital into order, and it was pleasant to find that the things, which had been all packed into one room, were perfectly dry. The little maid who helped in the hospital last year died during the winter, the only death in this place. There has, however, been much sickness, and one poor woman told us that she was ill for a long time, and had sat in bed and cried many a time because there was no doctor nor nurse to come and see to her and give her medicines.

On the 9th two of us went to Trap Cove and Mattie's Cove and visited the people, receiving welcomes which gave us much pleasure, they were so hearty. We heard all about the wonderful aches and pains they had experienced during the winter, and also of the death of two men. One of these was a Roman Catholic, and a woman said they were waiting for the priest to "come up long" to bury him. We asked what they had done with the body and she said, "Oh! he is underground;" so it was evidently the burial service over the grave which she was expecting the priest to undertake and not the actual interment. The winter has evidently been a very severe one out here, and a woman asked what the ice was like in our country when we started. She seemed very much surprised that icebergs should be novelties to us.

The Battle Harbour Hospital is now quite ready for occupation and everything looks very nice.

### Women Architects.

Lives there a woman who is wholly satisfied with her house, who does not think that if the builder had consulted her, she could have given him much practical advice? There is the insufficiency of cupboards, the absence of a convenient spot for a box-room, some incompleteness or malarrangement of the kitchen premises, and the bed-rooms with doors that ventilate and windows that don't. The average architect designs a house with good reception-rooms, a certain number of bed-rooms, good or bad, and the rest as the space at his command will allow. And the average woman enters the house because it is the best she can get, and grumbles all the time at the lack of domestic conveniences. But mere grumbling will not cure the evil, if only for the reason that no man will heed it. Given a comfortable chair, a fire, and a well-cooked dinner, and the man does not live who will rise up in wrath because his portmanteau spends its leisure under the bed, or the household linen occupies his wife's wardrobe. If you tell him there is no proper place in which to store the jam, he will calmly answer, "Don't make any; buy it at the stores as you want it," while any allusion to the absence of a laundry simply provokes the advice, "Send out the washing." Upon such advice the comment, "Yes; and have all the things burnt up with chemicals and rubbed to pieces with machinery," falls absolutely flat, while the additional remark, "I would require a larger housekeeping allowance" has been known to cause an attack of complete deafness. But instead of grumbling at that which is, why does not woman design her own house? We do not mean that every woman should be her own architect, but let some of the women who are now crying for an outlet for their energies enter an architect's office and go through a thorough course of training for this work. Let them learn how to use space wisely and economically, let them learn the cost and values of stone, brick, wood, and mortar, and let them add to that their practical feminine experience of household needs, and the lady architect may become one of the most useful and honoured of women workers; only let her not be too ambitious in her aim. A well-planned cottage is more to be desired than an ill-planned castle.

☞ For Everybody's Opinion, Reading to the Sick, Book World for Women and Nurses, &c., see page ccix. et seq.

### A Winter in the Sunny South.

By a Private Nurse.

Having finished a three years' training in a large London hospital, it was with mixed feelings of pleasure and regret that I went to the matron's office one morning. The matron looked up, and said, "Well, nurse, I am very sorry to lose you, but I do not wish to stand in your way. What are you thinking of doing?" I told her that I should like to get some nursing in the South of France, and she replied, "I think I can manage that for you."

After three months' rest, a course of instruction in massage, and a small amount of preliminary correspondence, a letter came one morning, saying that two well-known doctors on the Riviera were willing to engage my services. It was a welcome communication, for thoughts of wintering abroad were most congenial. At last the day for departure arrived, and, leaving Londoners ankle-deep in mud and slush, I journeyed towards Italian skies, enjoying all the details of my new experience.

From Paris, right on to Mont Cenis and Turin, the route lay through a perfectly white world of frozen cascades, terraces of icicles, and trees quite bridal in their attire, whilst brilliant sunshine transformed the country into a fairyland, which somehow inspired feelings of awe and reverence. Beyond Savona the whole scene was changed—the prevailing characteristic of the country being the hundreds and hundreds of orange trees, heavily laden with ripe fruit.

Enraptured with the blue of the Mediterranean, and of the sky and the brilliant sunshine, I began to think I had reached an enchanted land. Three hours after alighting from the train I was at a case, relieving the long night-watches with reflections on that wonderful journey. Next morning I witnessed a resplendent sun rise over the tideless sea in a haze of golden glory, with the faint outline of Corsica showing on the horizon.

The highway in this land of flowers on some of the Carnival days gave me another enjoyable and strange experience, which I stored in my memory, to be used when I returned to workaday life in the land of fogs and mud. After three months' constant nursing, principally diseases of the chest, there came a few days' leisure, which I gladly devoted to extending my knowledge of the Riviera. Monte Carlo (a little natural paradise) and its showy Casino, as well as the town of Nice, being visited.

Flowers of every sort, size, and description, in the greatest profusion, filled the air with delicious fragrance. Plants, only seen in England in hothouses, flourish in the open air of the Riviera.

Insect life also abounds freely; there are always plenty of flies and mosquitos. The latter get themselves very much disliked for their disfiguring ways, so the Riviera climate has its "cons" as well as "pros." At sunset there is a fall of temperature, which necessitates an invalid keeping indoors at that time, or else being provided with an additional thick wrap, to avoid the great danger of taking a chill.

The air is too exciting for many people, especially patients of a nervous type, so that rainy days are looked on, by physicians, as rather advantageous, rendering the air more sedative. The Mistral, winds blowing from the south-west, are very trying to everyone.

As the heat becomes more and more intense, so the list "des étrangers" becomes shorter and shorter, therefore the month of May found me almost as much excited about my return to England as I had been on my departure from it. I had an anxious journey back with an invalid, who, with valvular heart disease of many years' standing, had recently developed malignant disease of the liver. Beyond being detained three days at Calais by a rough sea, the journey happily came off without any grave inconvenience. I am still nursing the same case in one of the healthiest suburbs of London, and have been thanked by her friends and relatives for bringing her comfortably back to her native land. I found an early opportunity to revisit the scene of my "training" (which I shall always look back upon with much satisfaction, notwithstanding the arduous duties of hospital life), and to thank a much respected matron for assisting me in carrying out my wish to spend a winter in doing private nursing in the sunny South.

### Caithness.

The Duke and Duchess of Portland, who both take the keenest interest in the welfare of their employés and servants, have opened a cottage hospital for their benefit at Langwell, Caithness. It consists of a six roomed cottage, situated close to the sea, with a charming view of coast and river. The hospital is divided into two floors, two wards and a nurses' room being upstairs, and a day-room, kitchen, and laundry on the ground floor. All the walls are tinted in pretty pale colours and varnished, the floors being stained and polished. The beds with white quilts and crimson blanket folded across the feet, crimson screen, easy chair with cushion to match, white muslin curtains, and white framed pictures and texts upon the walls, leave a most pleasing impression on a visitor. The other ward has only one bed at present, but if required it would take two. The nurses' room, which is furnished comfortably, is situated between the two wards, and there is a well-stocked linen cupboard and all nursing requisites. The people in the neighbourhood were greatly interested in the furnishing and general arrangements of this charming cottage, one old body remarking, "Surely its just the cleanest place in Caithness." The hospital is intended for any case of serious illness or accident on the Duke's Caithness Estate.

### Minor Appointments.

Naples International Hospital.—Miss Katharine A. R. Fryer has been appointed extra English nurse at this hospital. She was trained at St. Saviour's Infirmary, East Dulwich.

The Mary Wardell Convalescent Home.—Miss A. M. Pepper, who was trained at St. Marylebone Infirmary, Notting Hill, London, has been appointed Sister at Miss Mary Wardell's Home at Stanmore.

### Notes and Queries.

The contents of the Editor's Letter-box have now reached such unwieldy proportions that it has become necessary to establish a hard and fast rule regarding Answers to Correspondents. In future, all questions requiring replies will continue to be answered in this column without any fee. If an answer is required by letter, a fee of half-a-crown must be enclosed with the note containing the enquiry. We are always pleased to help our numerous correspondents to the fullest extent, and we can trust them to sympathise in the overwhelming amount of writing which makes the new rules a necessity. Every communication must be accompanied by the writer's name and address, otherwise it will receive no attention.

**Queries.**

(116) *Dispensing.*—Where can I obtain information about dispensing for women?—*Lady Nurse.*

(117) *Annuity.*—Is there any association for helping trained nurses when past work, such as exist for governesses?—*Boscombe.*

(118) *Colonial.*—I shall be glad of information as to private nursing institution in Australia and at the Cape.—*Lois.*

(119) *Private Nurse.*—Can anyone with only one year's training and several years experience of private nursing hope to get a post as charge nurse in a general hospital?—*An Old Reader.*

(120) *Cripple.*—Can anyone tell me of a Home to which a crippled girl could be admitted by sending a million stamps.—*A Subscriber.*

**Answers.**

(116) *Dispensing* (*Lady Nurse*).—In Burdett's "Hospital Annual."

(117) *Annuity* (*Boscombe*).—You should write to the secretary of the Royal National Pension Fund for Nurses, 8, King Street, Cheapside, London, on the subject.

(118) *Colonial* (*Lois*).—Unless you take out plenty of introductions to doctors, hold very good certificates, and can afford to keep yourself for months when without work you had better not go abroad. The Colonies are training their own nurses very much now.

(119) *Private Nurse* (*An Old Reader*).—Not in a general hospital in London, possibly as assistant nurse in a workhouse infirmary.

(120) *Cripple* (*A Subscriber*).—We do not know of one.

### Erratum.

In the note headed "A Lesson for All," on page ccxi of the Nursing Supplement for the 11th inst., for "chloral" read "syrup of chloral" throughout.

### Everybody's Opinion.

[Correspondence on all subjects is invited, but we cannot in any way be responsible for the opinions expressed by our correspondents. No communications can be entertained if the name and address of the correspondent is not given, or unless one side of the paper only be written on.]

### THE MATRONS' COUNCIL.

"Qualified Nurse" writes: It certainly seems to me that "The Nurse Parliament" suggests itself as a better name for this scheme, although there must exist a marked difference between it and "the House," inasmuch as the members and associates are invited to discuss questions of general and professional interest, and not those of the nation. Modern culture has led to a professed scorn of the old-fashioned small talk of typical British matrons, children and servants forming their staple conversation, but hospital matrons, sisters, and nurses are now invited to be far more confidential, and to make confession to an "Advisory Committee" of their technical difficulties. It must be a comfort to outsiders to learn that the Matrons' Council "does in no way propose to interfere in the management of the hospital, or intervene between the authorities!" Is there, however, any matron who would like to think of her own subordinates criticising her management before a general meeting of past and present superintendents of British hospitals? And what sort of a position would such persons hold amongst their contemporaries in institutions afterwards? Any woman holding the important position of superintendent who finds she cannot manage her duties, aided and counselled by her committee and the medical staff (always willing to support a competent woman), assuredly ought to resign her post and go back into leading strings. "But surely you must find yourself sometimes in want of advice which only a woman can give you?" I once asked. "Of course I do," retorted the young clever matron then addressed; "but in that case I just think of the most experienced and best worker that I know and I lay the matter before her, and in five minutes everything is made clear, and the affair is never mentioned again between us. Gossip of professional matters to a committee of ladies, indeed! How long should I enjoy the confidence of my nursing staff, let alone the committee and the doctors, if I were guilty of such conduct as that?"

### PUBLICITY AND PROTECTION.

"Hibernia" writes: If publicity is the protection of paupers; as has recently been asserted in your paper, is it not true that it is even more essential to the well-being of lunatics—a still more helpless class? Can it be said to be afforded them while it continues to be the law (at least in Ireland) that it is not compulsory, should one of them be found dead in his bed, that an inquest should be held? Optional it is indeed with the relatives to demand one, but lunatics are often burdens—more or less an expense—a class "better off in a better world," and so relatives are or may be prone to avail themselves of what seems to be a slipshod ruling of the law which considers no inquest necessary when the patient has been under medical supervision, *i.e.*, that of the resident medical superintendent. Now what I want an opinion on is whether it is not throwing too much responsibility on, and placing too much temptation in the way of the said medical superintendent, that such should be the state of the law? He is only human—he wishes to be of good report, he desires promotion, he would fain avoid scandal and stand well in the eyes of his board, generally composed of non-professional men who might not understand his difficulties, and consider it a grave reflection on his management should a patient have his death accelerated by a push or blow from a fellow lunatic, or by a kick or "throttling" bestowed by an attendant—irritated by a troublesome case or excited by a potion of "John Jameson" of undue proportions. The ill-paid attendants of Irish asylums may not be miracles of patience or tact, but might they not be controlled and their behaviour regulated if inquests followed on sudden deaths as a matter of course, instead of, as now, being dependent on the consciences of medical superintendents or the whims of the relatives of the deceased lunatic? I hope this matter will be discussed in your columns, ever open to obtain justice for the helpless.

### For Reading to the Sick.

### THE TRUE SHEPHERD.

**Motto.**

The King of Love my Shepherd is.—*Sir Henry W. Baker.*

**Verses.**

Jesu, our Shepherd, cease not
Thy flock from harm to free,
And when Thy sheep are wandering,
O, lead them back to Thee. —*H. A. and M.*

I was wandering and weary,
When my Saviour come unto me;
For the ways of sin grew dreary,
And the world had ceased to woo me;
And I thought I heard Him say,
As He came along His way—
O silly souls! come near Me;
My sheep should never fear Me;
I am the Shepherd true!

At first I would not hearken,
And put off till the morrow;
But life began to darken,
And I was sick with sorrow;
And I thought I heard Him say,
As He came along His way—
O silly souls! come near Me, &c.

At last I stopped to listen,
His voice could not deceive me; . . . .
He took me on His shoulder,
And tenderly He kissed me;
He bade my love be bolder,
And said how He had missed me;
And I'm sure I heard Him say,
As He went along His way—
O silly souls! come near Me;
My sheep should never fear Me;
I am the Shepherd true!

**Reading.**

Of all the names by which our Lord Jesus Christ is called, not one is so touching, so beautiful, or so infinitely tender as that of a Shepherd.

The very word is full of peace. A picture rises up before the mind's eye of hills clothed with green, of sheep in happy contentment finding pasture, of a Shepherd in the midst with a look of love on His face, as He takes thought and care for His sheep. Read the following verses:—

". . . . He calleth His own sheep by name and leadeth them out."

"And when He putteth forth His own sheep, He goeth before them, and the sheep follow Him, for they know His voice."

"And a stranger will they not follow, but will flee from him, for they know not the voice of strangers."

"I am the Good Shepherd: The Good Shepherd giveth His life for the sheep."

"I am the Good Shepherd and know My sheep, and am known of mine."

"As the Father knoweth Me, even so know I the Father, I lay down My life for the sheep." "My sheep hear My voice, and I know them, and they follow Me."

No more beautiful words than these are in all the Bible: "He calleth His own sheep by name, and leadeth them out."

Here it seems as though tenderness had reached its height. . . . No longer am I one of a crowd; Jesus calls me by name, and, O wondrous thought! I may be His friend.

"My sheep hear My voice, and I know them, and they follow Me."

Dear Lord! do I hear Thy voice day by day? Thou speakest to me when my heart is very still, and when I read Thy Words in the Bible. But often I miss hearing Thy voice.

### The Book World for Women and Nurses.

[We invite Correspondence, Criticism, Enquiries, and Notes on Books likely to interest Women and Nurses. Address, Editor, The Hospital (Nurses' Book World), 428, Strand, W.C.]

Christina Chard. By Mrs. Campbell Praed. (London: Chatto and Windus.)

This is a novel which is little likely to add to Mrs. Campbell Praed's reputation either as an independent observer or as a creative genius. In "Christina Chard" there is no new departure in literary style, in scenic effect, in the grouping or interpretation of the characters; the authoress is the Mrs. Praed of a dozen other novels. Thus in the person of Christina Chard we are introduced to another goddess of beauty, the like of which can only be created by the prolific imagination of Mrs. Campbell Praed. The heroine has the same powers peculiar to the past favourites of the authoress, of enchanting and bewitching all sorts and conditions of men, from the brainless colonel to the hard-headed politician, and all the intermediate stages of imbecility and genius which can be included in an indefinite series of male individuals. Christina's papa runs a gold mine—in Leucharts Land, of course—floats a company in England, and sinks in it all the members of the above-mentioned indefinite series of male characters, except a few of the admirers of Christina, whom the latter is disposed to favour. Christina's powers of fascination are put to good account in avenging the sins of one individual on the whole of the male sex, of course with the above-mentioned selected exceptions. The sinner is a certain Sir Carr-Gambier, who appears to have sinned in a somewhat complicated manner. He first made love to Christina when she was young and penniless, married her, then left her, then denied that he had ever married her, and finally fell in love with her again, was married a second time, and is finally deserted himself. If the reader can keep his head quite clear through these complicated ramifications of the plot, there is much to amuse and interest, and on the whole he will not regret the trouble.

Lord Ormont and His Aminta. By George Meredith. (London: Chapman and Hall, Limited.)

The reader of George Meredith does not, or should not, expect to find meat for his delectation in the plot, neither does he anticipate to be holden or enthralled by thrilling situations or richness in incident. In these respects, however, the last of the Meredithean series will be a pleasant surprise to the less enlightened of his readers. We do not for an instant insinuate that "Lord Ormont and His Aminta" can be treated with the scant and superficial respect which the ordinary three-volume novel generally receives, and for the most part deserves. No; to enjoy or even understand the general design of the narrative—and this applies, perhaps, to a greater extent to the series of dialogues which strew the pages—the reader cannot afford to rush the perusal, much less to skip the less obvious *points d'appui*. Every sentence must be studied, construed, and analysed, and the reader may then return the volumes to the librarian with the self-satisfied knowledge that he has added to his mental archives, one of the most powerfully-written novels of the last decade. The history of Lord Ormont and his Aminta might have been written in two volumes, exacting critics might even put it at one volume, but as it is, George Meredith must have done some violence to his creed of rarification of incident when, in the short course of one novel, he allowed the curtain to rise to so many and so varied scenic effects. The story tells of a doughty warrior, Lord Ormont, and his capitulation to the childish fascinations of the beautiful but unknown Araminta. In so doing he offends against the aristocratic ambitions of his family in general and of his sister in particular. The latter, a stiff-backed and autocratic individual, hopes, as Society suspects, that this marriage, being contracted at a foreign embassy, is illegal and need not be recognised. Her hopes are, however, shattered, and Lord Ormont has to bewail the elopement of his Aminta with a young enthusiast of more suitable years. Lord Ormont's tacit admission of the justice of this step, in the concluding scene, affords possibly an unexpected, but perhaps none the less natural finale to a series of perfectly consistent situations. It is a novel for everyone to read once—many will not be satisfied with a single reading.

A Green Bay Tree. By W. H. Wilkins and Herbert Vivian. (Three vols., Hutchinson and Co.)

If "A Green Bay Tree" is not a success it will not be for want of advertisement. After a plenteous use of the puff preliminary, the appearance of the book was the immediate signal for the joint authors to quarrel, and air their grievances in print, a course which will doubtless have its effect upon the circulation of their volumes. Certainly, Mr. Wilkins' objection to being identified with Coryton is exceedingly natural, for a more unpleasant personage has seldom graced the pages of a novel. The aim of the story—if any aim there be—is, apparently, to show that honesty is not the best policy, and that vice in the person of Coryton may flourish Triumphant, while virtue, in the person of Tyrconnel, dies in a garret. The real attraction of the book, however, lies in reading between the lines and substituting names, more or less well-known, for the fictitious ones which the authors have bestowed upon their characters. This sort of puzzle game will doubtless amuse the friends and enemies of those held up to admiration or ridicule, as well as that large section of the community which gloats over the doings of the upper ten thousand in the society papers—but it is hardly literature. Neither is the elaborate system of indicating the exact portion of the book for which each of the authors is responsible, to be commended from the point of view of the reader; it is apt to be irritating, and interferes with the continuity of the story. The great majority of the reading public will probably vote "A Green Bay Tree" an exceedingly clever book, and if the principal ingredients of cleverness are cheap cynicism and conventional unconventionality, the majority of the reading public will be right in its verdict.

A Daughter of To-Day. By Mrs. Everard Cotes (Sara Jeannette Duncan). 2 Vols. (Chatto and Windus.)

Mrs. Everard Cotes, better known as Sara Jeannette Duncan, abandoning—not finally, we may hope—the lighter vein in which she has hitherto successfully worked, has broken new ground in her latest novel, "A Daughter of To-Day." This is a further contribution to the literature of the "Ewig Weibliche," but even those on whom the "Woman Question," as set forth in the novel of the period, is somewhat beginning to pall, must acknowledge the charm of Mrs. Cotes' keen insight and artistic treatment. The whole interest centres around the strange, essentially modern character of Elfrida Bell, the American girl, who, breaking free from restraint, sets off for Europe to "live her own life.' Unlovable, almost repellant, as she is at times, yet her pluck, her calm recognition of her mistakes, and her dogged determination to repair them, awaken a kind of sympathy and admiration, and even the minutest details of her career are interesting—each of them sheds some light upon the conflicting qualities that go to make up a strange, self-dependent character. The contrast between Elfrida and Janet Cardiff is an effective one, each woman seeming to be possessed of exactly the qualities which the other lacks. Most people, it is to be imagined, will vote Elfrida detestable, in spite of the fact that she is, to a great extent, a creature of circumstance; others, perhaps, will have a sneaking sympathy for her even in her most disagreeable moods; but, whatever be their opinion of her, very few, we imagine, will fail to be interested in her career.

